# A polymorphic transcriptional regulatory domain in the amyotrophic lateral sclerosis risk gene *CFAP410* correlates with differential isoform expression

**DOI:** 10.3389/fnmol.2022.954928

**Published:** 2022-09-05

**Authors:** Jack N. G. Marshall, Alexander Fröhlich, Li Li, Abigail L. Pfaff, Ben Middlehurst, Thomas P. Spargo, Alfredo Iacoangeli, Bing Lang, Ammar Al-Chalabi, Sulev Koks, Vivien J. Bubb, John P. Quinn

**Affiliations:** ^1^Department of Pharmacology and Therapeutics, Institute of Systems, Molecular and Integrative Biology, University of Liverpool, Liverpool, United Kingdom; ^2^Department of Psychiatry, National Clinical Research Centre for Mental Disorders, The Second Xiangya Hospital, Central South University, Changsha, China; ^3^Centre for Molecular Medicine and Innovative Therapeutics, Murdoch University, Perth, WA, Australia; ^4^Perron Institute for Neurological and Translational Science, Perth, WA, Australia; ^5^Department of Biostatistics and Health Informatics, Institute of Psychiatry, Psychology and Neuroscience, King's College London, London, United Kingdom; ^6^Department of Basic and Clinical Neuroscience, Maurice Wohl Clinical Neuroscience Institute, Institute of Psychiatry, Psychology and Neuroscience, King's College London, London, United Kingdom; ^7^NIHR Biomedical Research Centre, South London and Maudsley NHS Foundation Trust, King's College London, London, United Kingdom; ^8^Department of Neurology, King's College Hospital, London, United Kingdom

**Keywords:** *CFAP410*, ALS, VNTR, gene expression, transcriptional regulation

## Abstract

We describe the characterisation of a variable number tandem repeat (VNTR) domain within intron 1 of the amyotrophic lateral sclerosis (ALS) risk gene *CFAP410* (*Cilia and flagella associated protein 410*) (previously known as *C21orf2*), providing insight into how this domain could support differential gene expression and thus be a modulator of ALS progression or risk. We demonstrated the VNTR was functional in a reporter gene assay in the HEK293 cell line, exhibiting both the properties of an activator domain and a transcriptional start site, and that the differential expression was directed by distinct repeat number in the VNTR. These properties embedded in the VNTR demonstrated the potential for this VNTR to modulate *CFAP410* expression. We extrapolated these findings *in silico* by utilisation of tagging SNPs for the two most common VNTR alleles to establish a correlation with endogenous gene expression. Consistent with *in vitro* data, *CFAP410* isoform expression was found to be variable in the brain. Furthermore, although the number of matched controls was low, there was evidence for one specific isoform being correlated with lower expression in those with ALS. To address if the genotype of the VNTR was associated with ALS risk, we characterised the variation of the *CFAP410* VNTR in ALS cases and matched controls by PCR analysis of the VNTR length, defining eight alleles of the VNTR. No significant difference was observed between cases and controls, we noted, however, the cohort was unlikely to contain sufficient power to enable any firm conclusion to be drawn from this analysis. This data demonstrated that the VNTR domain has the potential to modulate *CFAP410* expression as a regulatory element that could play a role in its tissue-specific and stimulus-inducible regulation that could impact the mechanism by which *CFAP410* is involved in ALS.

## Introduction

Amyotrophic lateral sclerosis (ALS) is a fatal neurodegenerative disease primarily of the motor system, characterised by upper and lower motor neuron death, muscle atrophy and paralysis (Hardiman et al., 2017). ALS heritability has previously been estimated to be as high as 61%, indicating a significant genetic contribution to the variation in ALS risk and susceptibility (Al-Chalabi et al., 2010; Hardiman et al., 2017), and now there are over 40 genes associated with the disease, of varying effect sizes (Wroe et al., 2009; Abel et al., 2012; Shatunov and Al-Chalabi, 2021). Occasionally, patients can harbour multiple mutations, highlighting that ALS is a complex disease (van Blitterswijk et al., 2012a,b; Al-Chalabi and Visscher, 2014; Cady et al., 2015; Bury et al., 2016; Zou et al., 2017; Nguyen et al., 2018; Goldstein et al., 2019; Yilmaz et al., 2022). There is also evidence that ALS pathogenesis is a multistep process, with both genetic and environmental risk factors working in concert *via* gene–environment interaction (Al-Chalabi et al., 2014; Chio et al., 2018; Vucic et al., 2019; Garton et al., 2021). While genome-wide association studies have helped to uncover regions associated with ALS, it has been shown that single nucleotide polymorphisms (SNPs) only account for a small fraction of ALS heritability (van Rheenen et al., 2016). Furthermore, it has been shown that structural variants could be a key source of this missing heritability (Theunissen et al., 2020), with the hexameric repeat expansion in the first intron of the *C9orf72* gene the most commonly reported mutation in European ALS patients (Zou et al., 2017). Since the discovery of the *C9orf72* repeat expansion, there have been numerous studies investigating tandem repeat DNA and structural variation in ALS (DeJesus-Hernandez et al., 2011; Renton et al., 2011; Blauw et al., 2012; Lattante et al., 2018; Tazelaar et al., 2019, 2020; Course et al., 2020; Al Khleifat et al., 2022).

Tandem repeats are DNA sequence motifs that are recurrent and found contiguously in a region of the genome. Such tandem repeats constitute ~3% of the human genome (Bakhtiari et al., 2018; Hannan, 2018). They are often found to be polymorphic in the general population and are thus termed variable number tandem repeats (VNTRs); furthermore, specific alleles can be risk factors for diseases, which are often neurological in nature (Lam et al., 2018; Roeck et al., 2018; Pacheco et al., 2019). Non-coding VNTRs can be functional in a number of ways: they can act as transcription factor binding sites (Hsieh et al., 2007; Ali et al., 2010; Zukic et al., 2010), drive gene expression on the basis of repeat number (Warburton et al., 2015), work in concert with other genetic variants to induce combinatorial regulation (Warburton et al., 2016), regulate splicing (Roeck et al., 2018) and modulate gene expression and CpG methylation levels at the genome-wide level (Gymrek et al., 2016; Quilez et al., 2016). Thus, VNTRs are key transcriptional regulatory domains within the human genome, with repeat copy numbers driving differential gene expression profiles, altering affinity for transcription factors at gene promoters and modifying the secondary structure of DNA, all of which would modify gene expression both in a tissue specific and stimulus inducible manner (Marshall, 2021).

**Figure 1:**
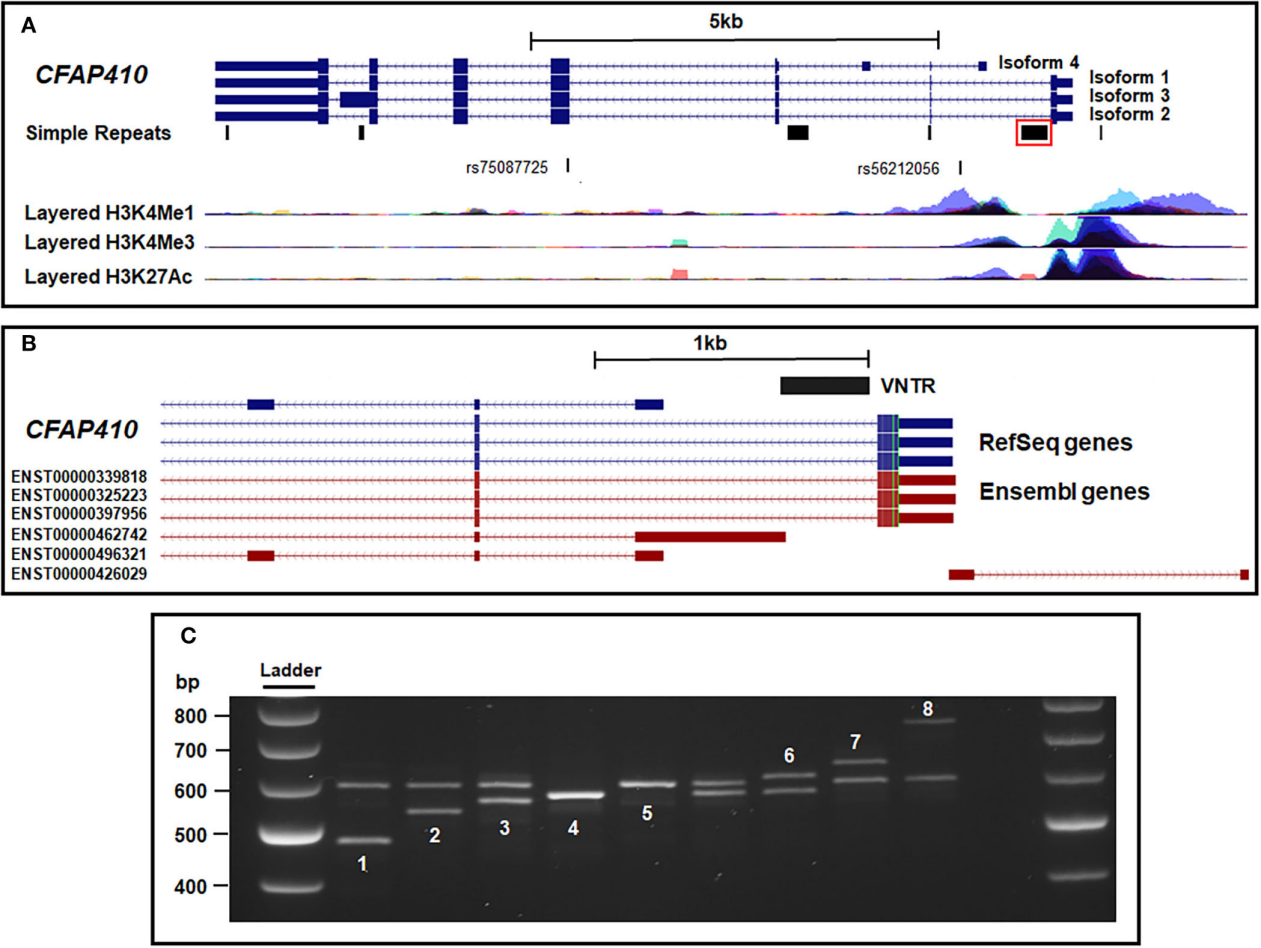
Location of multiple transcripts at the *CFAP410* gene locus relative to the intron 1 VNTR. **(A)** The four main isoforms of CFAP410 overlaid with the Simple Tandem Repeats track and histone marks from the ENCODE database. The ALS GWAS SNP (rs75087725) and identified tagging SNP (rs56212056) are also shown. The VNTR identified in this study is boxed in red. **(B)** *CFAP410* VNTR location overlaid with RefSeq genes from NCBI and predicted transcripts from Ensembl database. **(C)** PCR amplification and gel electrophoresis of the VNTR within the *CFAP410* locus. This region was confirmed to be polymorphic and eight alleles were identified (numbered 1–8 to reflect increasing length). Variant 7 was found only in a single ALS case. All samples were run at 100 V on 2% agarose for 3.5 h. All samples shown are from the cohort of ALS cases and controls that are genotyped in Table 1.

To date, five genes linked to ALS have been identified to contain tandem repeat domains (*C9orf72, ATXN1, ATXN2, NIPA1* and *WDR7*) demonstrating that VNTRs are a strong candidate for genetic risk for this disease (Sproviero et al., 2017; Iacoangeli et al., 2019; Tazelaar et al., 2019, 2020; Course et al., 2020). More recent global analysis to prioritise causal genes within ALS-risk loci has utilised not only rare pathogenic variants but also analysis of short tandem repeat domains of six bases or less (van Rheenen et al., 2021). This study highlighted the association of SNP rs75087725 within *Cilia and flagella-associated protein 410* (*CFAP410*) as a missense variant associated with ALS but found no evidence for other variants including short-tandem repeat expansions within this locus associated with ALS. However, the bioinformatic pipelines used would not be able to accurately address larger VNTRs. Our analysis of transcriptional regulation of the *CFAP410* gene highlighted six tandem repeats at this locus, five of which were not variable in repeat copy number. However, there was a VNTR in intron 1 of *CFAP410* with a complex repeat domain of 22 bp or 35 bp extending to 500–600+bp in length (Marshall, 2021) (Figure 1, Supplementary Figure 2B). We therefore tested the hypothesis that the *CFAP410* VNTR within intron 1 of the most 5' transcriptional start site is a transcriptional regulatory domain and that tandem repeat variation could be associated with both differential gene expression of *CFAP410* and risk for ALS.

## Materials and methods

### ALS cohort used for PCR genotyping of *CFAP410* VNTR

Genomic DNA purified from the blood of ALS cases and controls was obtained from the UK Motor Neuron Disease (MND) Collection DNA and Cell Bank, which was used for PCR-based genotyping of the *CFAP410* VNTR. A total of 500 ALS samples were provided, 456 from people fulfilling the El Escorial criteria, 21 with progressive muscular atrophy, 12 with ALS restricted to progressive bulbar palsy and 11 with primary lateral sclerosis. A total of 333 people with ALS were male and 167 were female, with an age range of 24–91 years old and a disease age of onset range of 23–88 years old. A panel of 499 controls was also obtained: 188 were male and 311 were female with an age range of 27–84 years old. The genotyping analysis was performed using this gender ratio because this was the total cohort size available to us in this database. We therefore did not generate a gender risk bias on the VNTR.

### Identification of the *CFAP410* VNTR

Tandem repeats in the *CFAP410* locus were identified on UCSC HG19 (https://genome.ucsc.edu/) using the Simple Tandem Repeats track from Tandem Repeat Finder (https://tandem.bu.edu/trf/trf.submit.options.html) (Benson, 1999). A total of six distinct tandem repeats were identified over this locus; we focused on the repeat within intron 1, which is the only one we found to be variable in repeat number.

### Genotyping the *CFAP410* VNTR

The VNTR was amplified by PCR, forward 5′ AACCCCAGACAACAGACCC 3′ and reverse 5′ CTGACGCGGAAGATGGTTC 3′ primers were designed to amplify the full-length VNTR sequence (584bp). Amplification reactions used KOD Hot Start DNA polymerase (Merck) with 1 × hot start buffer and the recommended protocol. The products were analysed on 2% agarose gels stained with ethidium bromide. Association analysis was performed comparing allele frequencies between controls and ALS cases using the software programme CLUMP with 100,000 simulations (Sham and Curtis, 1995).

### Cell culture

The human embryonic kidney cell line, HEK293 (ATCC^®^ CRL-1573™), was cultured in the Dulbecco's Modified Eagle's Media (DMEM) (Gibco) containing 4.5 g/L D-glucose and 200 mM L-glutamine (Gibco), supplemented with 10% foetal bovine serum (Gibco), penicillin/streptomycin (100 U/ml, 100 mg/ml; Sigma) and 1% (v/v) 100 mM sodium pyruvate (Sigma). Cells were incubated at 37°C in 5% CO_2_.

### Generation of *CFAP410* VNTR reporter gene constructs

The *CFAP410* VNTR was amplified from genomic DNA by PCR using the KOD Hot Start DNA polymerase following the recommended protocol. The VNTR was subcloned into the intermediate Zero Blunt PCR Vector (Invitrogen). This intermediate plasmid was then digested with restriction enzymes and the VNTR insert was cloned within the multiple cloning site of the reporter gene vectors either pGL3-promoter (pGL3P) or pGL3-basic (pGL3B) (Promega) in both orientations (forward and reverse) [restriction digests, vector maps and sequencing available at Marshall (2021)]. In pGL3P the VNTR was located upstream of the SV40 minimal promoter to address action as an activator, and cloned in pGL3B to address putative function as an inherent transcriptional start site.

### Cell transfection and dual luciferase assay

HEK293 cells were seeded at approximately 100,000 cells per well in 24-well-plates. After 48 h of incubation, cells were co-transfected with 1 μg reporter gene plasmid (firefly luciferase) containing the VNTR sequences and 20 ng pRL control vector for normalisation (Renilla luciferase; Novagen) using TurboFect™ transfection reagent (ThermoScientific/Fermentas), according to the manufacturer's protocol. TurboFect™ was removed after 4 h of incubation and replaced with fresh media. Luciferase activity of reporter constructs was measured 48 h post-transfection using a dual luciferase reporter assay system (Promega) according to the manufacturer's instructions.

### Generation of *CFAP410* VNTR tagging SNPs

Samples from the UK MND Collections DNA bank had previously been analysed as part of Project MinE (van der Spek et al., 2019), and therefore, SNP genotype data were available for individuals that were part of this study. The genotypes of SNPs located within 500 kb of the *CFAP410* gene were extracted from 232 individuals who were either homozygous or heterozygous for the two commonest alleles (4 and 5) of the VNTR. Analysis was performed using plink (v1.07) (Purcell et al., 2007) to identify SNPs in linkage disequilibrium (*r*^2^>0.7) with alleles 4 and 5 of the VNTR.

### Analysis of *CFAP410* isoform expression using NYGC ALS dataset

RNA-seq data from the Target ALS cohort obtained from the New York Genome Centre Consortium were analysed (https://www.targetals.org/research/resources-for-scientists/). Data were available from different tissues of 211 people, including 178 with ALS, 5 with other neurological diseases (frontotemporal dementia, Alzheimer's disease and multiple system atrophy), 2 with other motor neuron diseases (spinal bulbar muscular atrophy and polio) and 26 non-neurological controls (NNC). RNA-seq data from the following tissues (total 1,170) were included: cerebellum (161), frontal cortex (169), medial motor cortex (148), lateral motor cortex (147), unspecified motor cortex (9), occipital cortex (81), temporal cortex (10), sensory cortex (2), lumbar spinal cord (147), cervical spinal cord (161), thoracic spinal cord (76), iPS cell line (4), motor neuron cell line (4), choroid plexus (35), medulla (1) and liver (15) (Supplementary Table 1). Isoform quantification of RNA-seq data was performed using the Salmon tool (https://salmon.readthedocs.io). Salmon-generated quant files were imported into R using the *tximport* function from the *tximport* package of R (Soneson et al., 2015). Counts were extracted with the *DESeqDataSetFromTximport* function and raw counts were normalised using the median-of-ratios method, implemented in the *DESeq2* package (Love et al., 2014). The *DESeq2* package in R was also used to detect statistically significant differences in the *CFAP410* isoform expression profiles between different subject groups (ALS vs. NNC). The *ggplot2* package in R containing the *geom_boxplot* function was used to visualise the data specifying the *stat_summary* function to *mean*. The unpaired Wilcoxon test was used to compare two independent groups of samples and to demonstrate statistical significance (*p*-values and effect size can be found in Supplementary Figure 2A, and a histogram plot for the distribution of data for *CFAP410* ENST00000462742 is shown in Supplementary Figure 5).

Tagging SNP analysis identified that genotypes (*AA, GA* and *GG*) of rs56212056 are correlated with the two commonest alleles 4 (*A*) and 5 (*G*). The association of the three different SNP alleles with differential *CFAP410* isoform expression was analysed. The unpaired Wilcoxon test was used to compare two independent groups of samples and to demonstrate statistical significance between different cases and controls and tagging SNP genotype, respectively (*p*-values and effect size can be found in Supplementary Figure 2B). LD analysis of the *CFAP410* locus including the analysis of 31 SNPs from 243 individuals within Project MinE shows no evidence for significant LD with any of the tested SNPs across the region other than the associations made for alleles 4 and 5 of the *CFAP410* VNTR (Supplementary Figure 3).

## Results

### Characterisation of isoform expression at the *CFAP410* locus

The *CFAP410* gene contains an SNP (rs75087725) that is associated with ALS risk (*p* = 3.08 × 10^−10^) (van Rheenen et al., 2016). Analysis of the *CFAP410* gene using the tandem repeat track on UCSC (https://genome.ucsc.edu/) led to the identification of six distinct tandem repeat domains at this locus. We focus in this communication on the VNTR in intron 1 (Figure 1A), which was the only one that was variable in repeat number. We noted its location relative to the transcriptional start sites and its primary sequence had high GC content (84%) and sequences related to those found in the *C9orf72* VNTR (Supplementary Figure 1 and Figure 1B). It is difficult for global analysis such as ChIPseq to correctly assign ENCODE data over VNTRs themselves; however, when the VNTR was overlaid with ENCODE data, it was found to be adjacent to the markers H3K4Me1 (a marker of regulatory domains associated with enhancers), H3K4Me3 (a marker of regulatory domains associated with promoters) and H3K12Ac (a marker of active regulatory domains associated with active enhancer elements) (Supplementary Figure 1). On RefSeq analysis, this VNTR was found within intron 1 of isoforms 1–3 of *CFAP410* and it could be a component of a promoter region for isoforms that initiate 3' of the VNTR. Extending RefSeq analysis to include Ensembl transcript data increased the number of isoforms identified at this locus (shown in red in Figure 1A). Specifically, this identified a predicted transcript initiating immediately 5' of the VNTR, Ensembl transcript (ENST00000462742), and an anti-sense transcript relative to *CFAP410*, which was annotated as a long non-coding RNA on Ensembl (ENST00000426029).

### Expression of isoforms in CNS

**Figure 2:**
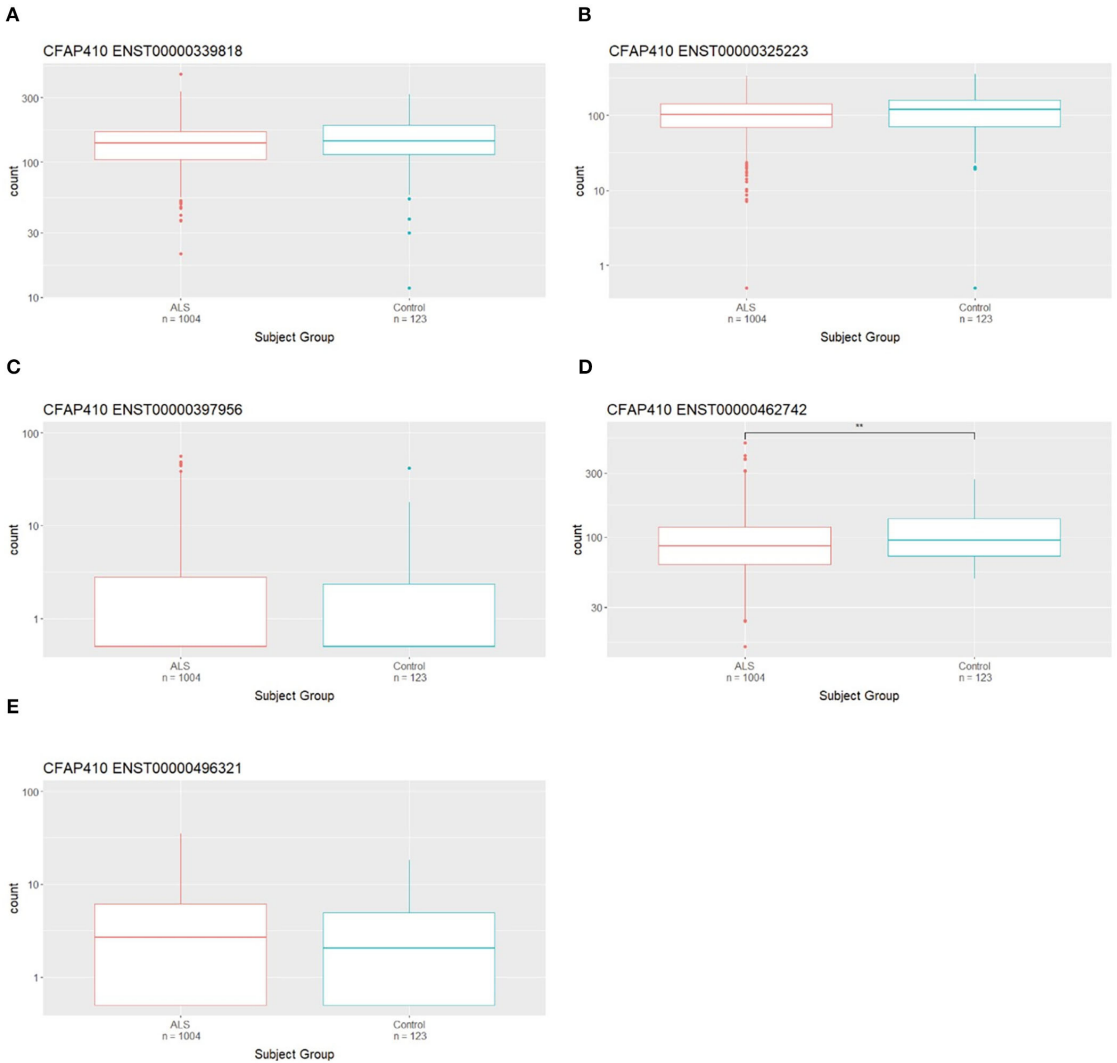
Differential expression of *CFAP410* in Case and Controls in the CNS using the NYG transcriptomic data. Expression analysis of *CFAP410* isoforms in ALS and control subjects. RNAseq data from the NYGC ALS cohort were used to compare expression of *CFAP410* isoforms ENST00000339818 **(A)**, ENST00000325223 **(B)**, ENST00000397956 **(C)**, ENST00000462742 **(D)** and ENST00000496321 **(E)**. Wilcoxon test was applied to demonstrate statistical significance indicated as asterisks. **P ≤ 0.01.

Transcriptomic data from the Target ALS cohort was utilised to assess the expression of *CFAP410* isoforms in the CNS. Isoform expression analysis showed that five *CFAP410* isoforms are expressed in the CNS (**Figure 5A**). The anti-sense transcript (ENST00000426029) relative to *CFAP410* showed no expression in this RNA-seq dataset (**Figure 5A**). We next analysed the differential expression of *CFAP410* isoforms in case and control subjects (Figure 2). The isoform ENST00000462742 showed significantly reduced expression in cases compared to controls (Figure 2D). This isoform was annotated as a non-coding retained intron transcript. The other two predominantly expressed isoforms (ENST00000339818 and ENST00000325223) demonstrated a similar pattern of expression to ENST00000462742; however, these associations did not reach statistical significance (Figures 2A,B). The remaining *CFAP410* isoforms (ENST00000397956 and ENST00000496321) showed very low expression levels, therefore the data for these isoforms did not give us sufficient power to identify evidence for differential expression between case and control subjects (Figures 2C,E).

### The intron 1 VNTR has a minimum of eight distinct alleles

PCR amplification and gel electrophoresis of the VNTR within the *CFAP410* locus was performed in a subset of the cohort from the UK MND Collection DNA Bank (*n* = 983). This region was confirmed to be polymorphic and eight alleles were identified (numbered 1–8 to reflect increasing length) (Figure 1C). The sequence of allele 5 of the VNTR (Supplementary Figure 1B) was found to be comprised of two distinct repeat lengths, 35 and 22bp long, respectively. Furthermore, four alleles were sequenced (Supplementary Figure 1B) and were found to contain the same two repeat lengths in varying patterns. From PCR of this limited number of genomes, the most common alleles were those termed 4 and 5, which contained 9 and 10 repeats, respectively (Supplementary Figure 1B).

### The *CFAP410* VNTR has both the properties of an activator domain and a transcription start site

**Figure 3:**
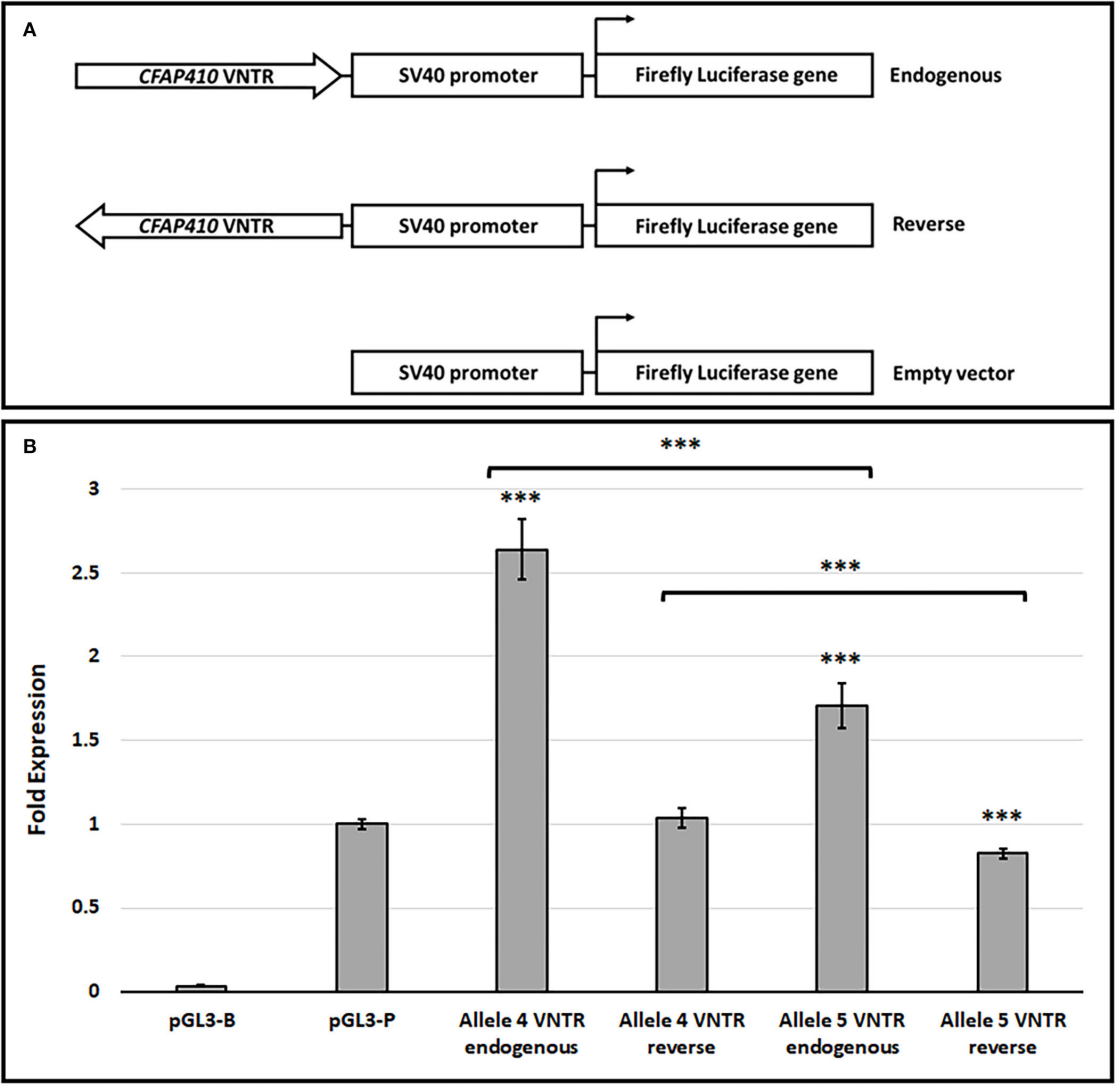
The *CFAP410* VNTR shows functional properties in the pGL3-P vector in the HEK293 cell line. **(A)** Schematic for VNTR containing constructs and pGL3 vectors (Promega) used in the luciferase assay. **(B)** The fold activity of alleles 4 and 5 of the *CFAP410* VNTR in the endogenous (forward) orientation within the pGL3P vector, normalised to the internal control Renilla luciferase (Biological replicate *n* = 3, technical replicate per assay *n* = 4). The promoter-less vector (pGL3-B) was included as a negative control. T-test was used to compare VNTR-containing constructs to SV40 unmodified vector (pGL3-P) and to compare all VNTR containing constructs against each other. ***P = <0.001.

To determine the potential ability of the *CFAP410* VNTR to regulate transcription, *in vitro* reporter gene assays were used. The most common alleles (alleles 4 and 5) were cloned in both orientations of the VNTR into the pGL3-P vector upstream of the SV40 minimal promoter and luciferase activity was compared to the activity of the pGL3-P vector alone. Both alleles demonstrated transcriptional regulatory properties (Figure 3). Alleles 4 and 5 in the endogenous orientation demonstrated a 2.64- and 1.71-fold increase in expression of luciferase over pGL3p alone, respectively, with both being statistically significant (Allele 5, 1.71 ± 0.13, *T*-test, *p* = 1.08 × 10^−6^ and Allele 4, 2.64 ± 0.18, *T*-test, *p* = 3.54 × 10^−6^). Furthermore, there was a significant difference in luciferase activity when cloned in this direction between alleles 4 and 5 (*T*-test, *p* = 4.39 × 10^−7^), with allele 4 showing a larger enhancement of reporter gene activity. In the reverse orientation, allele 5 alone demonstrated a mild decrease in expression compared to empty pGL3P (0.82±0.03, *T*-test, *p* = 1.49 × 10^−7^), but no change from empty pGL3P was observed for allele 4 in the reverse orientation (1.04 ± 0.06, *T*-test, *p* = 3.70 × 10^−1^).

**Figure 4:**
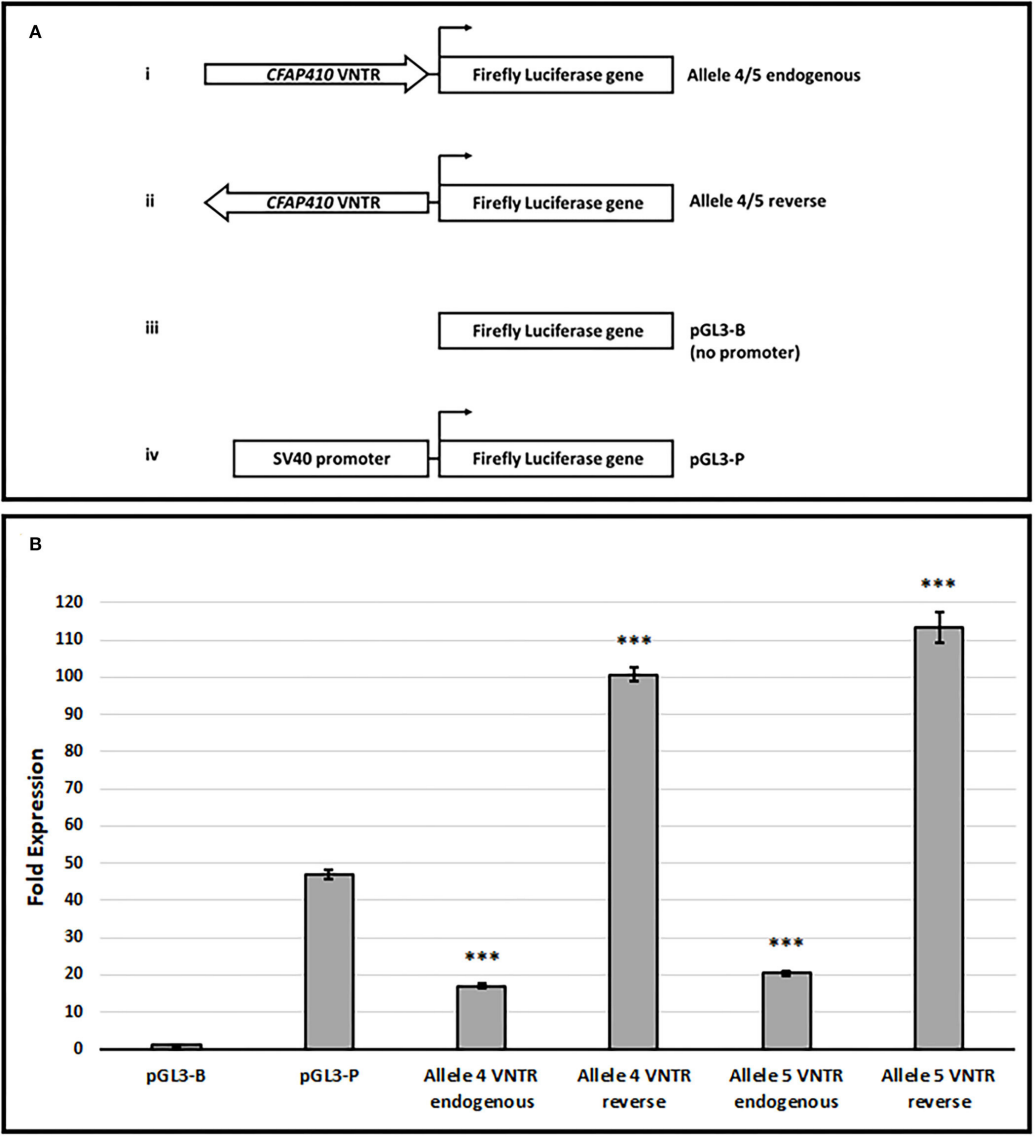
The *CFAP410* VNTR shows promoter activity in the pGL3-B vector in HEK293. **(A)** Schematic for VNTR containing constructs and pGL3 vectors (Promega) used in luciferase assay. **(B)** The fold activity of the *CFAP410* VNTR in the endogenous and reverse orientation within the pGL3-B vector normalised to the internal control Renilla luciferase (Biological replicate *n* = 3, technical replicate per assay *n* = 4). The pGL3-P vector was included as a positive control as this contains an SV40 promoter. *T*-test was used to compare VNTR containing constructs to each other (allele 4 vs. allele 5) and promoterless vector alone (pGL3-B). *T*-test was also used to compare VNTR containing constructs to the SV40 promoter vector (pGL3-P). ***P = <0.001.

The location of the VNTR upstream of the Ensembl transcripts ENST00000462742 and ENS00000496321 (equivalent position to the RefSeq short isoform) and the anti-sense transcript (ENST00000426029) (Figures 1A,B) suggested that it could elicit promoter activity. This might be especially true for ENST00000462742, which is directly adjacent to the VNTR, as it has been known for some time that GC-rich sequences can act to initiate transcription in those promoters that lack a TATA box (Kageyama et al., 1989). The VNTR primary sequence demonstrated high GC content (84%), which was consistent with such a model. To address this hypothesis, alleles 4 and 5 of the VNTR were cloned in both orientations into pGL3-B (Promega) containing a firefly luciferase reporter without a promoter, which is a well-characterised model to test if a region of DNA can act as a transcriptional start site in reporter gene studies. The unmodified pGL3-P vector (Figure 4) was included in this assay as it contains the minimal SV40 early promoter and therefore serves as a positive control for the level of expression directed by a well-characterised promoter in this cell line model. Both orientations of the VNTR demonstrated promoter activity which would require these domains to act as a transcriptional start site to allow expression of the luciferase reporter. This was especially true in the reverse orientation; VNTR orientations are given as endogenous and reverse to reflect that found in relation to the *CFAP410* gene. VNTR allele 4 in the endogenous orientation led to a 17.02-fold increase in luciferase expression (17.02 ± 0.67, *T*-test, *p* = 1.81 × 10^−13^), while the reverse orientation induced a 100.60-fold increase in reporter gene activity (100.60 ± 1.83, *T*-test, *p* =2.38 × 10^−17^) (Figure 4). Similarly, a 20.37-fold increase in luciferase expression was observed in the allele 5 endogenous construct (20.37±0.72, T-test, *p* = 4.88 × 10^−14^), while a 113.25-fold increase in luciferase activity in the allele 5 reverse orientation VNTR construct was observed (113.25 ± 4.16, *T*-test, *p* = 5.27 × 10^−14^). For comparison, the pGL3-P vector demonstrated a 46.98-fold increase in luciferase expression when compared to empty pGL3-B (46.98±1.28, *T*-test, *p* = 1.09 × 10^−15^). Thus, overall, a moderate but statistically significant increase in luciferase activity was observed in both endogenous orientation VNTR constructs when compared to empty pGL3-B, but this was significantly less than the SV40 minimal promoter activity seen with pGL3-P (*T*-test, *p* = 1.09 × 10^−15^). However, in the reverse orientation, a statistically significant increase compared to pGL3-P was observed: a 2.1- and a 2.4-fold increase in luciferase expression by alleles 4 and 5, respectively. Overall, both alleles of the VNTR induced a significant increase in reporter gene expression compared to empty pGL3-B, but allele 5 drove the largest effect in this model. This data suggests the possibility that the VNTR has promoter activity to initiate and direct expression at the *CFAP410* locus.

### VNTR variation is associated with *CFAP410* gene expression *in silico*

**Figure 5:**
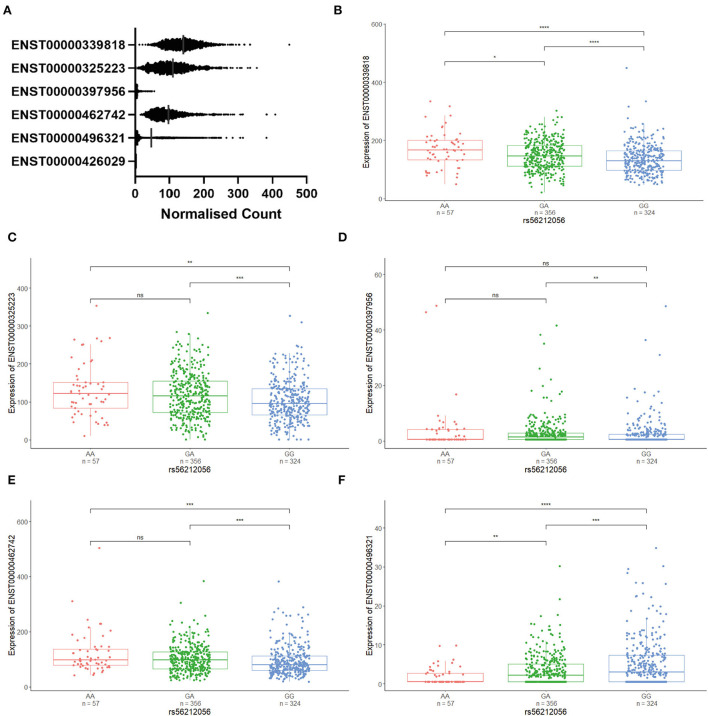
The expression of *CFAP410* isoforms is associated with tagging the SNP rs56212056 genotype. **(A)** CFAP410 isoform expression using RNAseq data from the NYGC Target ALS cohort. **(B–F)** Association of three different SNP genotypes (AA, GA, and GG) with an expression of isoforms ENST00000339818, ENST00000325223, ENST00000397956Q24, ENST00000462742, and ENST00000496321 was analysed based on the correlation with allele 4 (A) or allele 5 (G) using transcriptomic data from the NYGC ALS cohort. Wilcoxon test was applied to demonstrate statistical significance indicated as asterisks. *P = < 0.05, **P = < 0.01, ***P = < 0.001, ****P = < 0.0001, ns > 0.05.

We identified SNP rs56212056 to be in moderate linkage disequilibrium (*r*^2^ = 0.75, D' = 0.949) with the two most common alleles (termed alleles 4 and 5) of the VNTR. It was not possible to identify tagging SNPs for the rarer VNTR alleles in this study given their frequency in the population and the size of our cohort. The A allele of the SNP was found to be correlated with allele 4 of the VNTR and the G allele with allele 5. We used the Target ALS brain expression data to correlate these variants with *CFAP410* isoform expression. The association of three different SNP genotypes (*AA, GA* and *GG*) based on the correlation with allele 4 (*A*) or allele 5 (*G*) was analysed (Figure 5). We observed differential levels of isoform expression correlated with distinct G or A genotypes. Three *CFAP410* isoforms (ENST00000339818, ENST00000325223 and ENST00000462742) showed significantly reduced expression with *GG* genotype compared to both *GA* and *AA* genotype (Figures 5B-E). This was in contrast with the levels of expression of the isoform ENST00000496321 where the opposite pattern was observed (Figure 5E). Isoform ENST00000397956 correlated with significantly reduced expression in subjects with two copies of allele 5 (*GG* genotype) compared to subjects with the *GA* genotype only indicating one allele 4 and one allele 5 are present (Figure 5D). The data indicate that distinct VNTRs are able to support differential gene expression in the brain.

#### Genotyping the VNTR

To assess if specific VNTRs in this region were associated with ALS, the *CFAP410* VNTR was genotyped in a cohort of cases and controls (Table 1). The VNTR was amplified by PCR from blood-derived genomic DNA from patients (*n* = 490) and controls (*n* = 493) and a total of eight alleles of the *CFAP410* VNTR were identified and numbered according to increasing length. Allele 5 (reference genome variant, 584 bp) was the most common allele and was present in 74.49% of cases and 74.75% of controls; no significant difference in frequency was found between the two populations (Fisher's exact test, *p* = 0.75). The next most common variant was allele 4, which was identified in 22.86% of cases and 22.31% of controls: no significant difference in allele frequency was observed between the two groups (Fisher's exact test, *p* = 0.93). The other six alleles were infrequent in the population, with no statistically significant differences being observed (Table 1). There was also no significant difference in the allele frequencies of the eight alleles of the *CFAP410* VNTR when comparing controls and cases using CLUMP software (T_1_, *p* = 0.91 and T_4_, *p* = 0.94).

**Table 1:** Allele and genotype frequencies of *CFAP410* VNTR in MNDA cohort.

**(A)**							
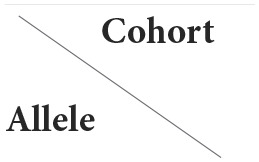	**ALS cohort**		**Control cohort**		**Total**	**% Difference (ALS–Control)**	***p*** **-value (Fisher's exact test)**
	**Count**	**%**	**Count**	**%**			
1	2	0.20	5	0.51	7	−0.30	0.67
2	3	0.31	2	0.20	5	0.10	0.50
3	10	1.02	11	1.12	21	−0.10	1.00
4	224	22.86	220	22.31	444	0.54	0.93
5	730	74.49	737	74.75	1,467	−0.26	0.75
6	5	0.51	6	0.61	11	−0.10	1.00
7	1	0.10	0	0	1	0.10	0.50
8	5	0.51	5	0.51	10	0.00	0.50
Total	980	100.00	986	100.00	1,966	0.00	N/A
**(B)**							
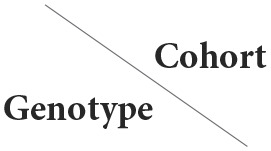	**ALS cohort**		**Control cohort**		**Total**	**% Difference (ALS - Control)**	***p*** **-value (Fisher's exact test)**
	**Count**	**%**	**Count**	**%**			
1.4	1	0.20	1	0.20	2	0.00	1.00
1.5	0	0.00	4	0.81	4	−0.81	0.22
2.2	1	0.20	0	0.00	1	0.20	1.00
2.4	1	0.20	0	0.00	1	0.20	0.50
2.5	0	0.00	2	0.41	2	−0.41	0.50
3.4	1	0.20	1	0.20	2	0.00	1.00
3.5	9	1.84	10	2.03	19	−0.19	0.71
4.4	28	5.71	27	5.48	55	0.24	0.57
4.5	161	32.86	159	32.25	320	0.61	0.59
4.6	3	0.61	3	0.61	6	0.00	0.50
4.8	1	0.20	2	0.41	3	−0.20	1.00
5.5	277	56.53	278	56.39	555	0.14	0.76
5.6	2	0.41	3	0.61	5	−0.20	0.61
5.7	1	0.20	0	0.00	1	0.20	0.50
1.8	1	0.20	0	0.00	1	0.20	1.00
5.8	3	0.61	3	0.61	6	0.00	1.00
Total	490	100.00	493	100.00	983	0.00	N/A

***(A)** The distribution of the eight identified alleles of the CFAP410 VNTR in an ALS cohort (n = 490) and matched controls (n = 493) was analysed. Alleles 2 and 7 were the least frequent, allele 7 being the rarest found only once in an ALS patient. There was no significant difference in allele frequency between the ALS cohort and matched controls (Fisher's exact test). (**B**) The distribution of the 12 identified genotypes of the CFAP410 VNTR in an ALS cohort and matched controls. There is no significant difference in genotype frequency between the ALS cohort and matched controls (Fisher's exact test)*.

To determine the repeat composition and length of the VNTR alleles, the two alleles found most commonly in this cohort (alleles 4 and 5) and two of the rare variants (alleles 2 and 8) were cloned and sequence validated. All alleles were found to be a composite of various numbers of both a 22 bp and 35 bp repeat unit (Supplementary Figures 1A,B). The primary sequence of the VNTR demonstrated variation in the actual sequence of both the 22 or 35 bp repeat unit and the repeating structure indicated a more complex evolution of variation than simply separating into two blocks. It may be of interest to note that the most 5' repeat element had only 34 bp.

## Discussion

We have demonstrated that the VNTR in intron 1 of the *CFAP410* gene was functional as a transcriptional regulator *in vitro*. It served as a transcriptional regulatory domain in reporter gene constructs in the HEK293 cell line and directed differential gene expression on the basis of repeat number as both an activator domain and transcriptional start site/promoter. These data were extended to *in silico* analysis in which tagging SNP analysis for the two most common alleles of this VNTR was associated with differential gene expression of *CFAP410* in the brain. This VNTR was found to be highly variable in the general population, with at least eight distinct variants, but there was no significant association of specific genotype with ALS risk. Nevertheless, our data indicated the regulatory properties of this VNTR could support differential regulation of *CFAP410* based on the copy number of the repeat. The ability of the VNTR to direct such expression could be one parameter that works in conjunction with other mechanisms at this locus that could lead to the progression or severity of ALS to specific challenges in the CNS.

It has been established that VNTRs can be both biomarkers for disease risk and also functional regulatory domains affecting both transcription and post-transcriptional mechanisms as reviewed recently (Marshall et al., 2021). These latter transcriptional properties can be exhibited together for the same VNTR as in the case of the monoamine oxidase A (*MAOA*) gene (Manca et al., 2018) and a VNTR in the mir137 gene (Warburton et al., 2016). Indeed, we have previously demonstrated that a human-specific intron 2 VNTR within the serotonin transporter gene can direct both tissue-specific and differential levels of reporter gene expression in a mouse transgenic model (MacKenzie and Quinn, 1999). We hypothesise that similar mechanisms may operate on the *CFAP410* VNTR, which could lead to distinct levels of expression from different alleles in response to the same cellular challenge. Specifically, we theorise that the association of the two most common alleles with differential *CFAP410* expression can be extrapolated to such properties being embedded in the other minor VNTR alleles.

The genomic location of the *CFAP410* gene is a complex region with multiple isoforms that can be either coding or non-coding in nature, indeed one of the isoforms ENST00000462742 has the hallmarks of a retained intron (Monteuuis et al., 2019) (Figure 1). Interestingly, this isoform was significantly downregulated in the cases relative to the controls. Notably, retained introns have higher GC content compared to introns that are not and this is true for the large GC-rich VNTR found in this intron of *CFAP410*, similarly the difference in the size of the VNTR and thus relative GC content could affect the efficiency of retention. This is consistent with a model in which the presence of RNA structures induced by the GC-rich microsatellite expansions has an inhibitory effect on splicing (Sznajder et al., 2018). New techniques and improved sequencing protocols have demonstrated as much as 95% of the genome can be transcribed, hence alternative splicing and intron retention are emerging areas of importance for gene regulation and disease progression (Monteuuis et al., 2019). In addition to the aforementioned *CFAP410* isoforms, there is also a non-coding RNA that is antisense to the *CFAP410* gene (Figure 1); however, we could find no evidence for measurable levels of this antisense message in the CNS transcriptomic data.

Genotyping the *CFAP410* VNTR led to the discovery of eight alleles in the cases and controls used for this study (*n* = 983) (Figure 1C). The frequencies of all alleles and genotypes were not found to be significantly different between cases and controls (Table 1); however, our limited cohort size would likely to be insufficient to identify rare variants associated with ALS. ALS is a complex disease and given the small sample size we were unable to address many of the different metrics, which contribute to this disease, including disease severity, the onset of disease, disease pathology, age, ethnicity, and known ALS genetic mutations in these patients: the VNTR in question could be important in any one of these metrics. However, we do pose a potential mechanism as to how such variants could contribute to disease risk and show that tandem repeat numbers can drive differential gene expression. Furthermore, the original *CFAP410* ALS association was from a rare variant (van Rheenen et al., 2016), so such rare VNTR variants discovered here could be part of a yet unidentified polygenic risk score.

Unfortunately, expansion of the cohort size is both time- and resource consuming due to the requirement for PCR analysis of all samples. However, the properties of the VNTR to modulate gene expression *in vitro* and correlation with differential *CFAP410* expression *in silico* allowed us to hypothesise that this domain could regulate the expression of this locus in response to the same challenge, which could result in distinct levels of protein expression that could modulate risk and progression of ALS. The primary sequence of the *CFAP410* VNTR also contains consensus sequence binding sites for proteins predicted to bind the *C9ORF72* intronic VNTR (Supplementary Figure 4), potentially inferring contribution to the same signalling pathways. Similarly, modulation of the levels of *CFAP410* could have a significant effect on the function of other ALS risk genes, such as functional interactions with *NEK1* (Fang et al., 2015; Watanabe et al., 2020). These studies demonstrate the increased fusion of genetic risk with an environmental challenge to modulate ALS risk and progression.

## Data availability statement

The original contributions presented in the study are included in the article/Supplementary material, further inquiries can be directed to the corresponding author.

## Ethics statement

The studies involving human participants were reviewed and approved by Trent Research Ethics Committee in February 2003 ref MREC/02/4/107 and in July 2009, ref 09/HO405/32. Please see https://www.ncbi.nlm.nih.gov/pmc/articles/PMC4501191/ for more details. The patients/participants provided their written informed consent to participate in this study.

## Author contributions

JM, AF, VB, and JQ: study idea and design. JM, AF, LL, BM, TS, AP, SK, VB, and JQ: data analysis and interpretation. JM: manuscript drafting. All authors reviewed and approved the final manuscript.

## Funding

JM was funded by a Medical Research Council Doctoral Training Studentship [Grant Number MR/N013840/1]. AF is a recipient of a Andrzej Wlodarski Memorial Research Ph.D. scholarship. Motor Neurone Disease Association (Grant Number: 41/523) supports the work of VB and JQ. AF, VB, and JQ by the Andrzej Wlodarski Memorial Research. BM, VB, and JQ are supported by the Darby Rimmer MND Foundation. LL was supported by the Chinese Scholarship Council 201806370099. SK and AP were supported by MSWA and Perron Institute. AA-C is an NIHR Senior Investigator (NIHR202421), also supported by an EU Joint Programme—Neurodegenerative Disease Research (JPND) project grant through the following funding organisations under the aegis of JPND–www.jpnd.eu[United Kingdom, Medical Research Council (MR/L501529/1; MR/R024804/1) and Economic and Social Research Council (ES/L008238/1)] and through the Motor Neurone Disease Association, My Name'5 Doddie Foundation and Alan Davidson Foundation. This study represents an independent research part funded by the National Institute for Health Research (NIHR) Biomedical Research Centre at South London and Maudsley NHS Foundation Trust and King's College London. This work was supported by resources provided by the Pawsey Supercomputing Centre with funding from the Australian Government and the Government of Western Australia.

## Conflict of interest

The authors declare that the research was conducted in the absence of any commercial or financial relationships that could be construed as a potential conflict of interest.

## Publisher's note

All claims expressed in this article are solely those of the authors and do not necessarily represent those of their affiliated organizations, or those of the publisher, the editors and the reviewers. Any product that may be evaluated in this article, or claim that may be made by its manufacturer, is not guaranteed or endorsed by the publisher.

## Supplementary material

Supplementary Figure 1 **(A)** *CFAP410* VNTR repeat unit breakdown. Number of 22 bp and 35 bp repeat units per allele of the *CFAP410* VNTR (confirmed through Sanger sequencing). **(B)** *CFAP410* VNTR alleles primary sequence and repeat order. Variants 2, 4, 5 and 8 of the *CFAP410* VNTR aligned and split into respective 22 bp and 35 bp repeat units. Allele 2 = 262 bp, allele 4 = 297 bp, allele 5 = 319 bp, allele 8 = 468 bp. Boxes indicate exemplars of the 35p and 22 bp repeat unit. All repeats have been aligned by eye and are therefore arbitrary.

Supplementary Figure 2 **(A)** P-values and effect size of analysis of *CFAP410* isoform expression using NYGC ALS dataset. **(B)** P-values and effect size of tagging SNP analysis of *CFAP410* VNTR.

Supplementary Figure 3 LD plot over *CFAP410* locus. LD plot over a 45kb region using 31 SNPs from 243 individuals from the Project MinE encompassing the *CFAP410* gene. Two blocks of LD are located 5' and 3' of the *CFAP410* gene. The ^*^ indicates rs56212056 which is the SNP in moderate LD with allele 4 and 5 of the VNTR located in *CFAP410*. There is minimal LD between rs56212056 and the other SNPs located in the region. Intensity of each square represents the r^2^ between the two SNPs; darker the colour the larger the r^2^.

Supplementary Figure 4 Alignment of *CFAP410* and *C9ORF72* intronic VNTRs. Both VNTR sequences are polymorphic and contained within intron 1 of the most 5' start site. The hexamer sequence within *C9orf72* from the UCSC browser **(A)** contains the sequence CCCCGG and GCCCCG identified in red and green respectively which are also found in the CFAP410 sequence **(B)**.

Supplementary Figure 5 Histogram plot of the distribution of data in the Target ALS cohort for the *CFAP410* ENST00000462742 transcript.

Supplementary Table 1 Available tissues from the NYGC Target ALS cohort stratified by controls and ALS subjects.

## Abbreviations

ALS: Amyotrophic lateral sclerosis
ENCODE: The Encyclopaedia of DNA Elements
CFAP410: Cilia and flagella associated protein 410
MAF: Minor allele frequency
MND: Motor neuron disease
NNC: Non-neurological controls
NYGC ALS: New York Genome Centre Consortium Target ALS
SNP: Single nucleotide polymorphism
UCSC, University of California: Santa Cruz
VNTR: Variable number tandem repeat.
