# Supplementary figures and images for: A polymorphic transcriptional regulatory domain in the amyotrophic lateral sclerosis risk gene CFAP410 correlates with differential isoform expression
Source: Front Mol Neurosci. 2022 Sep 5;15:954928. doi: 10.3389/fnmol.2022.954928 (PMC9484465; doi:10.3389/fnmol.2022.954928)

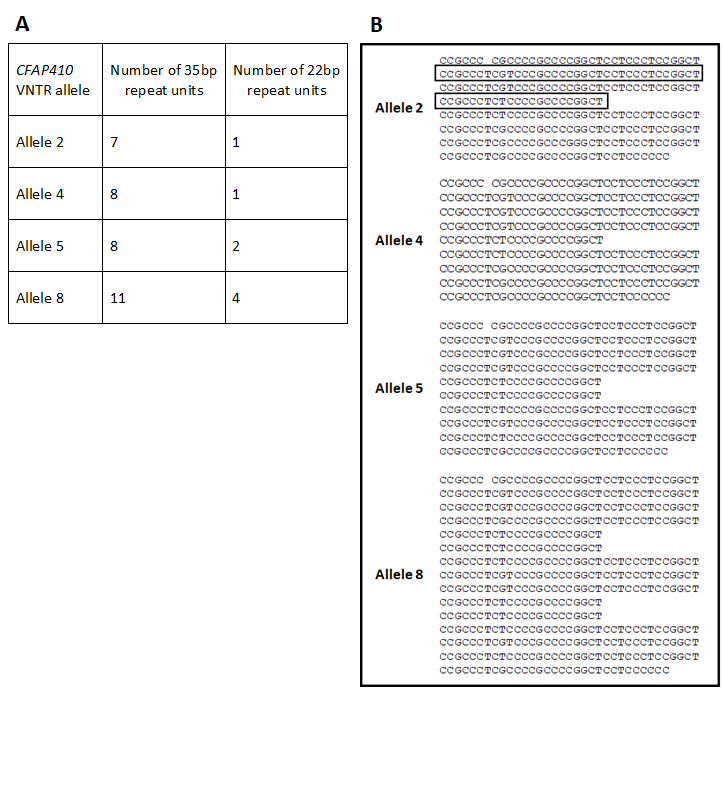

Supplement: Supplementary Figure 1 — (A) CFAP410 VNTR repeat unit breakdown. Number of 22 bp and 35 bp repeat units per allele of the CFAP410 VNTR (confirmed through Sanger sequencing). (B) CFAP410 VNTR alleles primary sequence and repeat order. Variants 2, 4, 5 and 8 of the CFAP410 VNTR aligned and split into respective 22 bp and 35 bp repeat units. Allele 2 = 262 bp, allele 4 = 297 bp, allele 5 = 319 bp, allele 8 = 468 bp. Boxes indicate exemplars of the 35p and 22 bp repeat unit. All repeats have been aligned by eye and are therefore arbitrary. [file Image_1.tif]

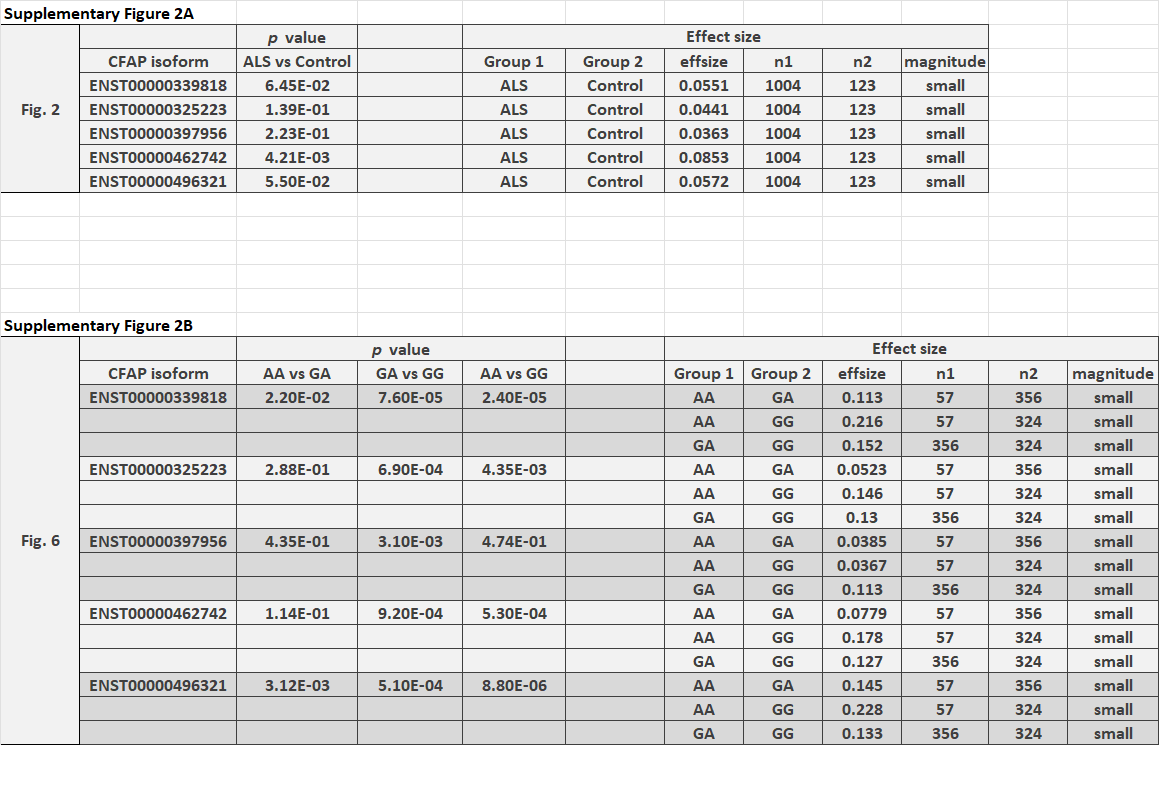

Supplement: Supplementary Figure 2 — (A) P-values and effect size of analysis of CFAP410 isoform expression using NYGC ALS dataset. (B) P-values and effect size of tagging SNP analysis of CFAP410 VNTR. [file Image_2.tif]

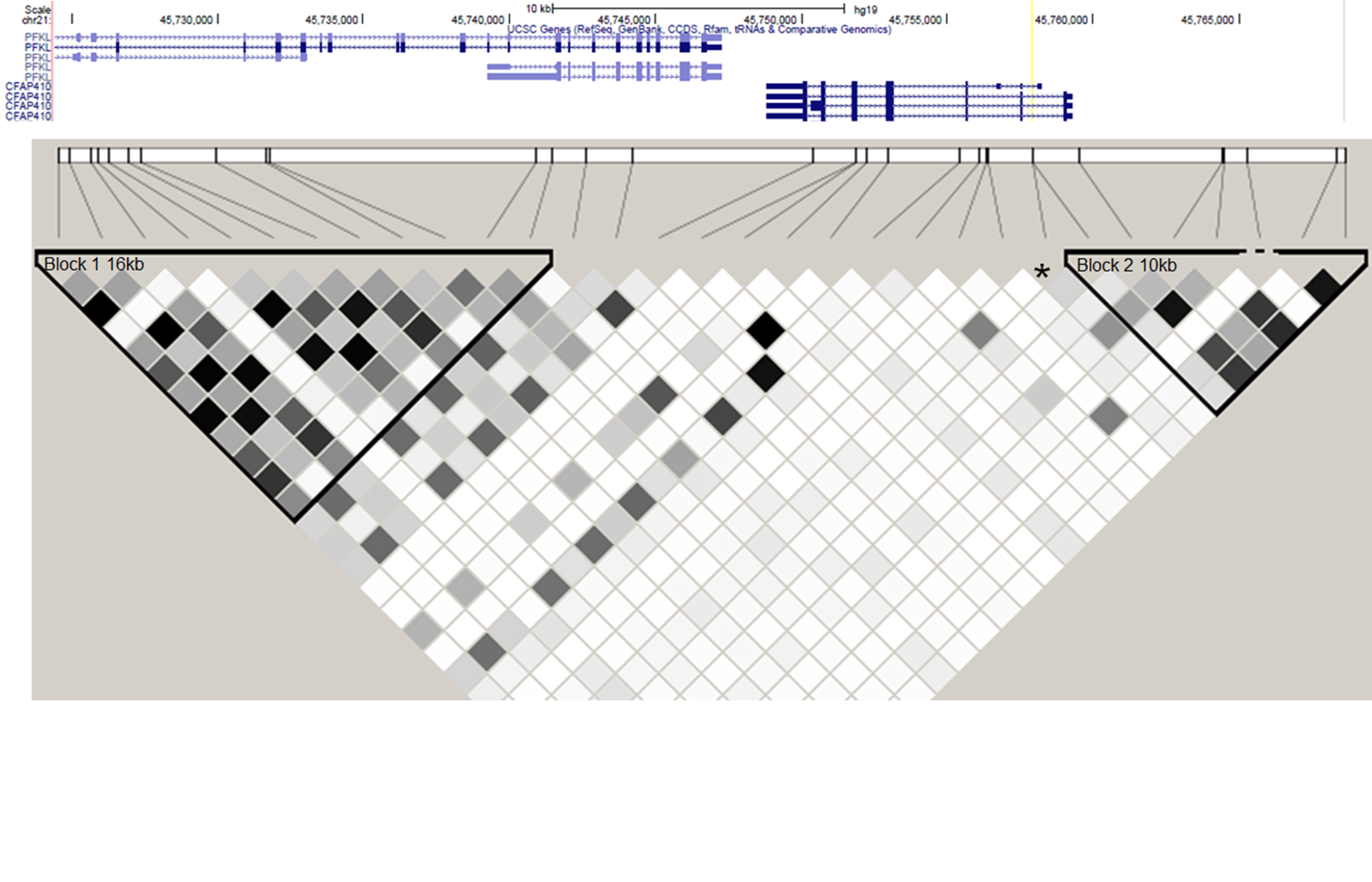

Supplement: Supplementary Figure 3 — LD plot over CFAP410 locus. LD plot over a 45kb region using 31 SNPs from 243 individuals from the Project MinE encompassing the CFAP410 gene. Two blocks of LD are located 5' and 3' of the CFAP410 gene. The * indicates rs56212056 which is the SNP in moderate LD with allele 4 and 5 of the VNTR located in CFAP410. There is minimal LD between rs56212056 and the other SNPs located in the region. Intensity of each square represents the r2 between the two SNPs; darker the colour the larger the r2. [file Image_3.tif]

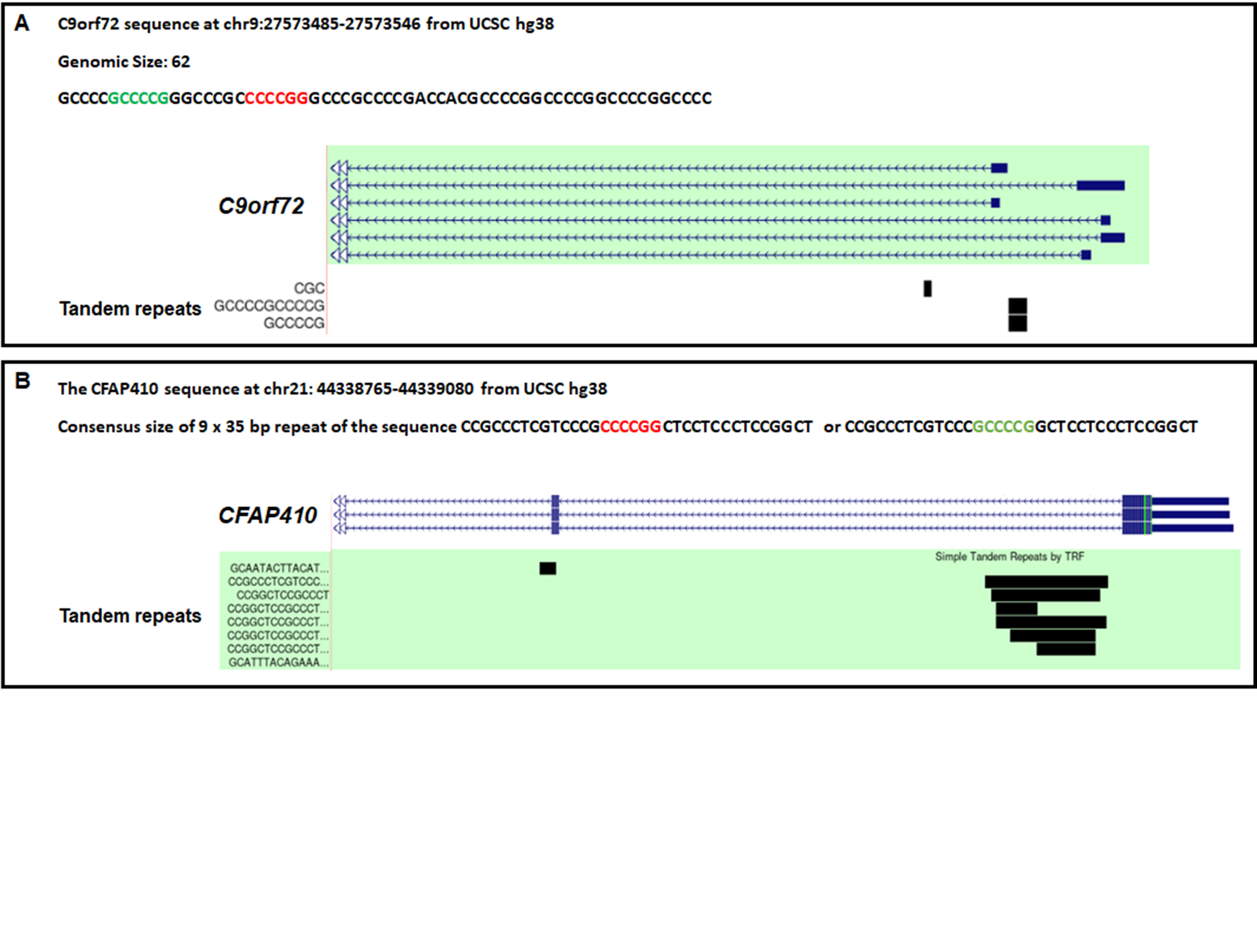

Supplement: Supplementary Figure 4 — Alignment of CFAP410 and C9ORF72 intronic VNTRs. Both VNTR sequences are polymorphic and contained within intron 1 of the most 5' start site. The hexamer sequence within C9orf72 from the UCSC browser (A) contains the sequence CCCCGG and GCCCCG identified in red and green respectively which are also found in the CFAP410 sequence (B). [file Image_4.tif]

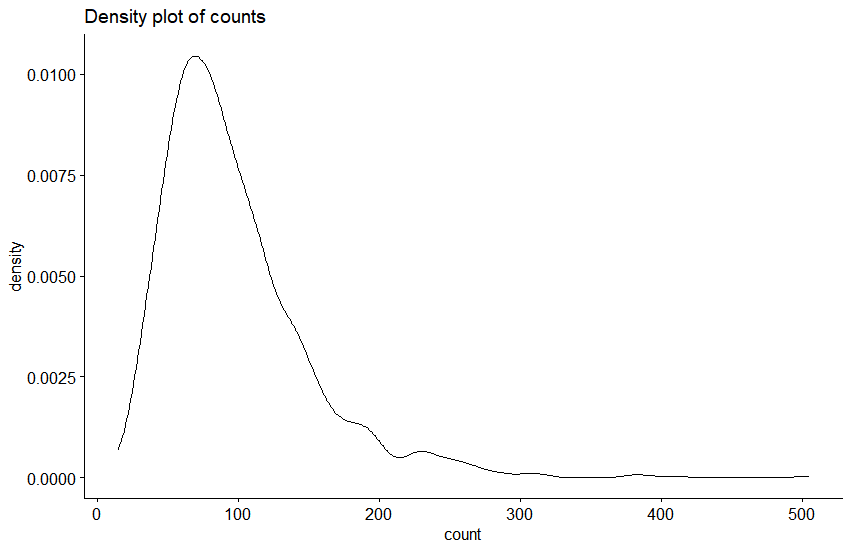

Supplement: Supplementary Figure 5 — Histogram plot of the distribution of data in the Target ALS cohort for the CFAP410 ENST00000462742 transcript. [file Image_5.tiff]
