# Supplementary material for: A polymorphic transcriptional regulatory domain in the amyotrophic lateral sclerosis risk gene CFAP410 correlates with differential isoform expression
Source: Front Mol Neurosci. 2022 Sep 5;15:954928. doi: 10.3389/fnmol.2022.954928 (PMC9484465; doi:10.3389/fnmol.2022.954928)
Supplement: Supplementary Table 1 — Available tissues from the NYGC Target ALS cohort stratified by controls and ALS subjects. [file Table_1.docx]

|  | Control | ALS |
| --- | --- | --- |
| MEDIAL MOTOR CORTEX | 18 | 124 |
| LATERAL MOTOR CORTEX | 14 | 128 |
| UNSPECIFIED MOTOR CORTEX | 1 | 8 |
| FRONTAL CORTEX | 18 | 146 |
| Temporal Cortex | 1 | 9 |
| Sensory CORTEX | 0 | 2 |
| OCCIPITAL CORTEX | 7 | 70 |
| CERVICAL SPINAL CORD | 16 | 139 |
| LUMBAR SPINAL CORD | 13 | 129 |
| THORACIC SPINAL CORD | 9 | 63 |
| Cerebellum | 13 | 141 |
| Choroid | 6 | 29 |
| Liver | 1 | 13 |
| MOTOR nEURON CELL LINE | 3 | 1 |
| IPS cell line | 3 | 1 |
| Medulla | 0 | 1 |
| TOTAL | 123 | 1,004 |

**Supplementary Table 1.** Available tissues from the NYGC

Target ALS cohort stratified by controls and ALS subjects.
